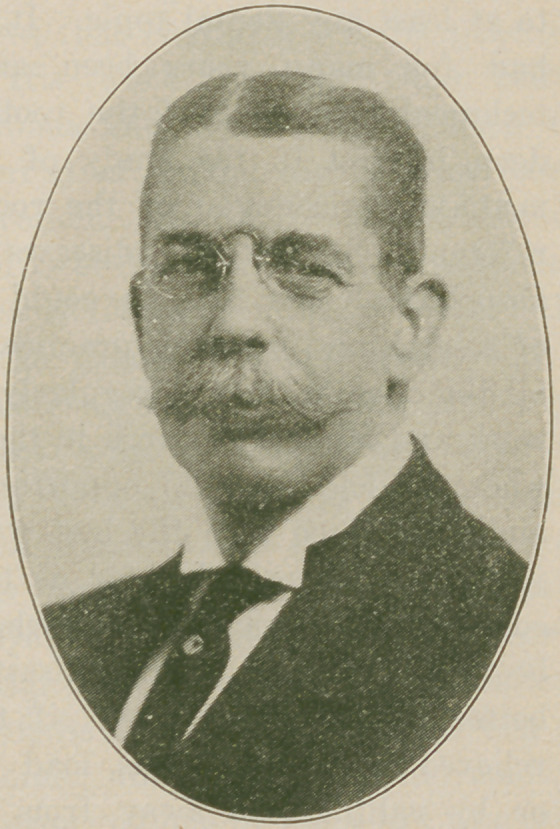# Event and Comment

**Published:** 1918-03

**Authors:** 


### DENTAL REGISTER

Vol. LXXII MARCH, 1918 No. 3

### EVENT AND COMMENT

**Dr. John R. Callahan** We are again called upon to record the passing away by death of one who has for many years occupied a prominent place in the technical, literary and professional activities of our profession. Dr. Callahan was found dead in his bed at the Queen City Club of Cincinnati, where he was spending the night after a hard day's work in his office. The coroner found he had died from cerebral hemorrhage. His physician had many times cautioned him about his strenuous devotion to his practice and the extra professional work he was carrying but he always said there were so many things he wanted to do that he had to take the risk of a health break down. It is a sad end to a fine career, but it has so often happened that we don't seem to be able to learn by the experience of others. This life is altogether too short for even the brainiest men to work out their ideals in, and so we crowd on the work and speed up the machine until the break comes.

Dr. Callahan had been working for years on one of the most vital problems which the dental profession has to solve. Vital because it directly concerns the health and welfare of humanity almost as much as any other disease that afflicts mankind. It is only within the past five years that the relations of disease of the periapical region of teeth to general health has seriously attracted the attention of the medical and dental professions, but today it is undergoing the most critical study in all its relations by both professions that has ever been given to at least any dental topic. It was because Dr. Callahan had for many years been an ardent worker in the technical treatment of the root canals of teeth that his deep interest in the study of the pathology of the immediate environment of the roots of teeth, with especial reference to systemic diseases became his hobby and absorbed all the time he could get from the busy hours of a large and exacting practice. He studied his problem all the time, and gave regularly of his time to laboratory work and clinical research in the great Cincinnati hospital where he had splendid opportunities for clinical material and conference with the best workers on the related subjects. It is impossible for us to estimate how great and constant this strain was or what its influence may have been in bringing on the disastrous result. How much better it would have been if the profession could have relieved this tremendous load by giving him a helper, or by calling him away from practice entirely so that he could have worked out the problem for the benefit of us all. But we did not, as is usually the case, realize that our friend was working at such disadvantage and with such sincere devotion that he lost sight of his own welfare that he might bring health and comfort to humanity. It is not for us to censure anyone for wrongdoing, but it does seem that in view of the fact that has been demonstrated over and over again in our own profession, and now once again, we should begin to realize that the men in our profession who are giving their very lives, as well as their personal interests to the solution of the serious problems of our profession should deserve more than our post mortem adulations. It may be a wise plan for us to see to it that some of our other research men whom we can't spare are more carefully guarded, as they are all too few. Dr. Callahan will be greatly missed in the councils of the dental societies where he was faithful to his obligations in the same way and with the same success that characterized his practice and scientific work. His work for the Ohio State Society has been continuous and effective, and much of the success of this organization during the past has been due to his untiring devotion. He was a genial friend, and a host of hearts all over the land will be saddened by the news of what seems like an untimely death. He has left his mark on the pages of dental history in a very definite and laudable contribution to better dentistry, and future work on root canal treatment will have to take account of the work he contributed to the cleansing and hermetical sealing of root canals. Dr. Callahan was the son of a physician and lived and took his early training in Hillsboro, Ohio. He took his dental degree in the Philadelphia Dental College in 1877. He practiced for two or three years in San Francisco and returned to Hillsboro where he remained until 1889 when he removed to Cincinnati, where he acquired one of the select practices of that city. He has had many honors of a professional character bestowed upon him. The most significant perhaps was the bestowal by the New York State Dental Society upon him of the Wm. Jarvie medal for his contributions to dental science, which he received last June.

Dr. Callahan is survived by his widow, an unmarried daughter, a married daughter living in Detroit and a married son living in Phoenix, Arizona.

**Oklahoma Society Post Graduate School**

We have received the announcement of the annual meeting of this society and are impressed with the seriousness and enterprise of the profession as is manifest in the preparations they are making for this meeting. With a membership of less than three hundred they propose to hold a six days' meeting and import five instructors on the livest topics of the day. Each of these instructors is to spend all of the time with one section of the members as a class. This will be some task for the instructor, but he should be able to give his class a fairly good idea of his methods in that time. The question which occurs to us is whether this method is necessary to enable the better posted dentists to acquire a working knowledge of any technical procedure. It would seem that five full days of instruction on any of the subjects would be rather tiresome, even though it is proposed that the class do some part of the work, very much as college students do in the clinical courses. If enough of that kind of instruction is included to give each dentist an opportunity to put into practice the instruction given, the time may be all too short for definite results. It would seem that the time of the dentists would be conserved and the instructors' physical strength better conserved if the attending dentists could take at least two courses instead of one. As we read the announcement the course is prepared for the general practitioner and not for the specialist and it would seem that some provision should be made so that the general practitioner could at least hear the lectures of all the instructors and then be permitted to give all his spare time to the practical work in which he is specially interested. We can see no difficulty in arranging a work schedule so that everyone could at least have the privilege of the didactic instruction, and then divide into classes for the individual or clinical instruction. Even if more time was required in which to present the course it would accomplish more for the general practitioner than by the plan under discussion. Such a plan would keep the general practitioner up to date in general practice and at the same time would enable him to get started in a better method on at least one subject each year.

## Figures and Tables

**Figure f1:**